# Nerve growth factor responsive elements modulate immune cell inflammation and are dysregulated in an Alzheimer’s disease mouse model

**DOI:** 10.3389/fimmu.2026.1722477

**Published:** 2026-04-23

**Authors:** Ruchi Gera, Giuseppe Mocci, Simone Tambaro, Michael Vanlandewijck, Per Nilsson, Maria Eriksdotter, Sumonto Mitra

**Affiliations:** 1 Department of Neurobiology, Care Sciences and Society, Division of Clinical Geriatrics, Center for Alzheimer Research, Karolinska Institutet, Huddinge, Sweden; 2 Department of Medicine, Huddinge, Karolinska Institutet, Huddinge, Sweden; 3 Department of Neurobiology, Care Sciences and Society, Division of Neurogeriatrics, Center for Alzheimer Research, Karolinska Institutet, Stockholm, Sweden; 4 Department of Immunology, Genetics and Pathology, Uppsala University, Uppsala, Sweden; 5 Theme Inflammation and Aging, Karolinska University Hospital, Huddinge, Sweden

**Keywords:** Alzheimer’s disease, cholinergic, immune cells, inflammation, nerve growth factor, neuroimmune-communication, neurotrophin

## Abstract

Inflammation is a crucial regulator of body’s defense mechanism primarily modulated by the immune system. Context dependent immune activation (e.g., pathogen) requires acute inflammatory reactions followed by efficient resolution of inflammation. Impaired resolution may lead to chronic inflammation, often associated with several pathological processes, including dementia disorders. At present, the mechanisms of inflammation resolution are poorly understood. High levels of the neurotrophin nerve growth factor (NGF) are evident at the site of inflammation, however the effect of NGF on immune cells is debated, ranging from proinflammatory to anti-inflammatory. Thus, identifying the immune cells which possess NGF responsive receptors is crucial to understand how NGF can modulate immune function of those specific immune cells. Utilizing multi-color flow cytometry, we mapped across various immune cell subtypes including adaptive and innate immunity landscape from unchallenged mouse spleen for the presence of NGF receptors (TrkA and p75). Although NGF receptors were previously reported in some immune cell types, we report comprehensive cell-type dependent expression of NGF receptors on immune cells including dendritic cells, macrophage, natural killer (NK) cell as well as various subsets of lymphocytes (T and B cells). Since NGF is the upstream regulator of cholinergic signaling, we employed single cell RNA sequencing (scRNA-seq) and observed heterogenous neurotrophin-cholinergic landscape among various immune sub-sets, and report discordance between RNA and protein level expression. Interestingly, we found that differential activation methods ex-vivo could increase TrkA and p75 protein levels differently, a crucial regulator of NGF downstream signaling. Furthermore, we report that NGF supplementation reduced inflammatory cytokine production in activated T- and B-cells significantly. Using a mouse model of AD, we show age-dependent alterations in TrkA and p75 in immune cells, indicating altered NGF-immune coupling in AD. In conclusion, this study identifies that immune cells are direct recipient of NGF signaling by expressing its associated receptors and the existence of a novel inflammation regulatory mechanism mediated through NGF-receptors. The NGF responsive elements get hampered in the immune cells in an AD mouse model which may have pathogenic implications in the resolution of chronic inflammatory response. Thus, NGF associated mechanisms constitute novel regulatory potential in immune cells which can be targeted for inflammatory immune disorders.

## Graphical Abstract

**Figure:**
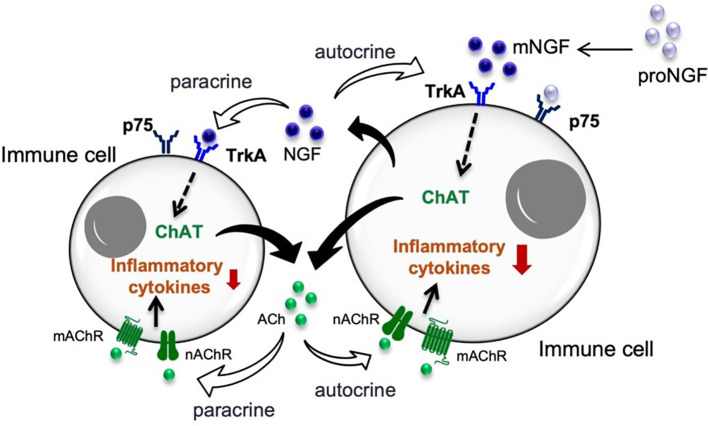
Proposed model of NGF-TrkA signalling in immune cells: We show that immune cells express TrkA and p75 receptors, possess cholinergic features through expression of ChAT (49), and furthermore could receive cholinergic signal through expression of ACh receptors (54) – representing a complete interactive system, although a large heterogeneity of gene and protein expression is observed. The NGF-TrkA interaction is known to activate various downstream pathways including the expression of ACh producing enzyme ChAT, thereby initiating the cholinergic signaling pathways (55). Thus, an additional regulatory loop is present in ChAT expressing immune cells wherein they produce ACh which may activate cholinergic receptors in an autocrine (if these cells also express ACh receptors) or paracrine (on adjacent cells expressing ACh receptors) manner. Thus, immune cells have a self-sustaining cholinergic machinery through NGF-TrkA-ChAT-ACh loop, which may play a role in autoregulation of inflammatory pathways and perhaps immune function. This is further strengthened by the fact that some immune cells (macrophage and CD4^+^ T cell) produce NGF themselves (56, 57), presenting a feed-forward loop of NGF-cholinergic machinery.

## 1 Introduction

Immune cells are a part of various processes ranging from physiological maintenance to inflammatory reactions including injuries and infections. Under physiological conditions, inflammation plays a crucial role in the immune response during infection or injury (1), but uninterrupted inflammatory reactions may lead to undesired chronic inflammation which is linked to various diseases ranging from cardiovascular to autoimmune diseases and neurodegenerative disorders (2). Thus, resolution of inflammation is equally important to prevent chronic inflammation-induced diseases. The human body has evolved mechanisms to control abrupt inflammatory reactions and combat chronic inflammation, via cell dependent and independent processes of inflammation resolution (3–5). Among the cell independent mechanisms, two of the well-known processes are the cholinergic anti-inflammatory pathway (CAIP) and the resolution of inflammation through lipid mediators (4, 6). CAIP is reported to occur via the vagal nerve induction, leading to the release of norepinephrine in the peripheral nerve endings, which further stimulate locally available immune macrophages to induce the production of the cholinergic neurotransmitter acetylcholine (ACh) which further acts on T cells to reduce inflammatory cytokine production (7, 8). Potential spatial limitation may occur primarily due to localized cholinergic nerve endings within various tissues including the spleen, as well as due to the rapid clearance of ACh from the soluble phase by the immensely active cholinesterase’s (AChE, acetylcholinesterase; and BChE, butyrylcholinesterase) (9). The lipid mediators, however, are produced by various cell types and are rather unspecific in distribution (10, 11). Recently, a body-brain circuit which modulates inflammation has received considerable attention wherein communication of the central nervous system with peripheral physiology is reported (12), alongside effects of peripheral processes on brain health (13).

Since immune cells are major producers of several classes of inflammatory cytokines, a regulatory mechanism could ideally be within the immune cells which can be targeted to induce an anti-inflammatory phenotype. Earlier reports have described such a type of mechanism in few immune cell types, describing receptors for the neurotrophic ligand nerve growth factor (NGF) or other classes of cholinergic receptors, but their involvement and occurrence on most immune cell types are unknown (14–17). Inflammatory stimuli were reported to induce NGF production from fibroblasts, endothelial, epithelial and glial cells but limited knowledge exists on the effect of NGF on immune cells at the inflammatory site (17, 18). NGF, well-known as the survival factor of cholinergic neurons in the brain, binds to its receptor TrkA (Tropomyosin receptor kinase A) which initiates choline acetyltransferase (ChAT) enzyme activity, thereby controlling the production of ACh from cholinergic neurons. NGF is also known to interact with another receptor p75 (low-affinity nerve growth factor receptor, NGFR or CD271) on neurons which is associated with inflammation and cell death and serves as a common receptor to engage other neurotrophins including NT-3, NT-4/5 and brain-derived neurotrophic factor (BDNF) (19).

In this study, we evaluate whether specialized immune cells possess NGF responsive elements expression, or it is a systemic phenomenon. We delve into the various types and sub-types of immune cells and probed for the presence of NGF related receptors – TrkA and p75. To check whether these pathways are biologically functional in immune cells, we studied whether immune activation modulates NGF responsive markers and if NGF can induce anti-inflammatory effect. Recent studies have highlighted the significant involvement of immune system in Alzheimer’s disease (AD) (20) and the contribution of inflammatory responses in AD pathology (21). Since NGF metabolism is affected in the brain and plasma of AD patients (22, 23), we investigated whether the components of the NGF-associated pathway in immune cells are affected using an amyloid beta (Aβ) pathology mice model of AD.

## 2 Materials and methods

### 2.1 Animals

In this study, female wild-type (WT) C57BL/6J (from Janvier labs, France) and *App^NL-G-F^* knock-in (NLGF) mice were used. NLGF mice contain the Swedish (KM670/671NL), Arctic (E693G), and Beyreuther/Iberian (I716F) mutations in the humanized Aβ peptide, which develops Aβ plaques in the brain from the age of 2 months onwards, with cognitive deficits from 6 months onwards (24). Mice were kept on a 12h light/dark cycle and food and water were available *ad libitum*. Mice were deeply anesthetized using isoflurane to collect spleen. Dissected spleens were processed to collect splenic immune cells as described in the cell culture section below. All experiments were performed in accordance with approved ethical permits ID 407 approved by the Linköping animal ethical committee and 12.570–2021 and 5406–2020 approved by the Stockholm animal ethical committee.

### 2.2 Cell culture

Splenocyte isolation: Single cell suspension from dissected spleens were obtained by mechanically dissociating the minced organ through a nylon mesh (100-micron; BD Biosciences) using RPMI (Cat no. A1049101, Invitrogen, USA). Cells were centrifuged at 1500 rpm for 15 min and collected cell pellet was treated with RBC lysis buffer (BD biosciences, USA) for 5 min at 37°C. Following 15 min centrifugation at 1500 rpm, collected splenocytes was resuspended in complete RPMI containing 10% FBS (Cat no. 0010, Hyclone, USA). Splenocyte suspensions were either processed for flow cytometry or single cell sequencing or cultured ex-vivo.

Ex-vivo culture of splenocytes: Splenocytes (1×10^6^ cells per well) were plated in 96 well plate and stimulated with PMA (100 ng/ml; Phorbol myristate acetate; Cat no. P8139, Sigma) and ionomycin (2 μg/ml, Cat no. I0634, Sigma) or LPS (1 μg/ml, Cat no. 437650, Sigma) for 48hr and collected to examine TrkA and p75 expression by flow cytometry. For experiments involving NGF treatment, seeded splenocytes were challenged with PMA-ionomycin followed by treatment with mature-NGF (Alomone labs;100ng/ml) for 48hr. After Golgi stop (Cat no. 554724, BD biosciences, USA) treatment during the last 4hr, splenocytes were processed for analysis of TNF-α cytokine by Flow cytometry.

### 2.3 Flow cytometry

Splenocytes were stained to identify various immune cells including B cell, T cell, macrophage, dendritic cell, natural killer cell and subsets of T cell using fluorochrome conjugated antibodies against various cell surface and intracellular molecules. Briefly, splenocytes were surface stained with various fluorochrome conjugated antibodies at 4°C for 15 min. Surface-stained cells were fixed and permeabilized using Foxp3 transcription factor fixation/permeabilization concentrate and diluent (Cat no. 00-5523-00, eBiosciences, USA) for 20 min at 4 °C followed by intracellular staining at 4 °C for 30 min against various intracellular molecules. Information for all antibodies are listed in the **Supplementary Table S1**. Samples were acquired on a LSR II flow cytometer (BD Biosciences) and data analysed on FlowJo software (version 10.8.1) was exported to GraphPad prism 10 software.

### 2.4 10X genomics single-cell gene expression sample processing and sequencing

After isolation, the splenocytes from C57BL/6J mouse were counted and the viability was measured with the Luna FL dual fluorescence cell counter with dual stain Acridine Orange/Propidium Iodide, with cell size gated between 1-60 μm (Logos Biosystems). Cell suspensions with viability of 85% were encapsulated using the Next Gem Single Cell 3′ Reagent kit v3.1. (Dual index) from 10x Genomics (cat: PN-100026), aiming to capture 5,000 cells. Libraries were prepared as per manufacturer’s instruction (protocol CG000315) and sequenced using the Illumina platform on a Next seq2000 P3–100 cycles flow cell, aiming for an average read depth of 40,000 reads/cell.

The sequencing data were demultiplexed and aligned using the cell ranger pipeline v 6.1.2 (10X genomics) using the GRC38 mm10 mouse genome build. The cell capture, library generation and data analysis were performed at the Single Cell Core Facility of Flemingsberg Campus (SICOF) that receives funding from the Infrastructure Board at Karolinska Institutet.

### 2.5 Gene-signature analysis

For single cell data analysis, the Seurat package version 4.0.1 for R version 4.0.5 was used. Clustering analysis was done by PCA reduction in 30 dimensions, and the cluster generation was performed with a resolution of 0.5.

### 2.6 Statistical analysis

All statistical analysis, except the analysis of scRNA-seq data were performed using GraphPad Prism 10 software. Data were analysed by one-way or two-way ANOVA with Tukey correction for multiple comparisons or unpaired *t-*test as per the statistical purpose and the number of groups. Results are presented as means ± SD in the bar plots and significance levels are *p < 0,05, **p < 0,01, ***p < 0,001, **** p < 0,0001.

## 3 Results

### 3.1 Immune cells possess NGF-responsive machinery through TrkA and p75 expression

**Figure 1:**
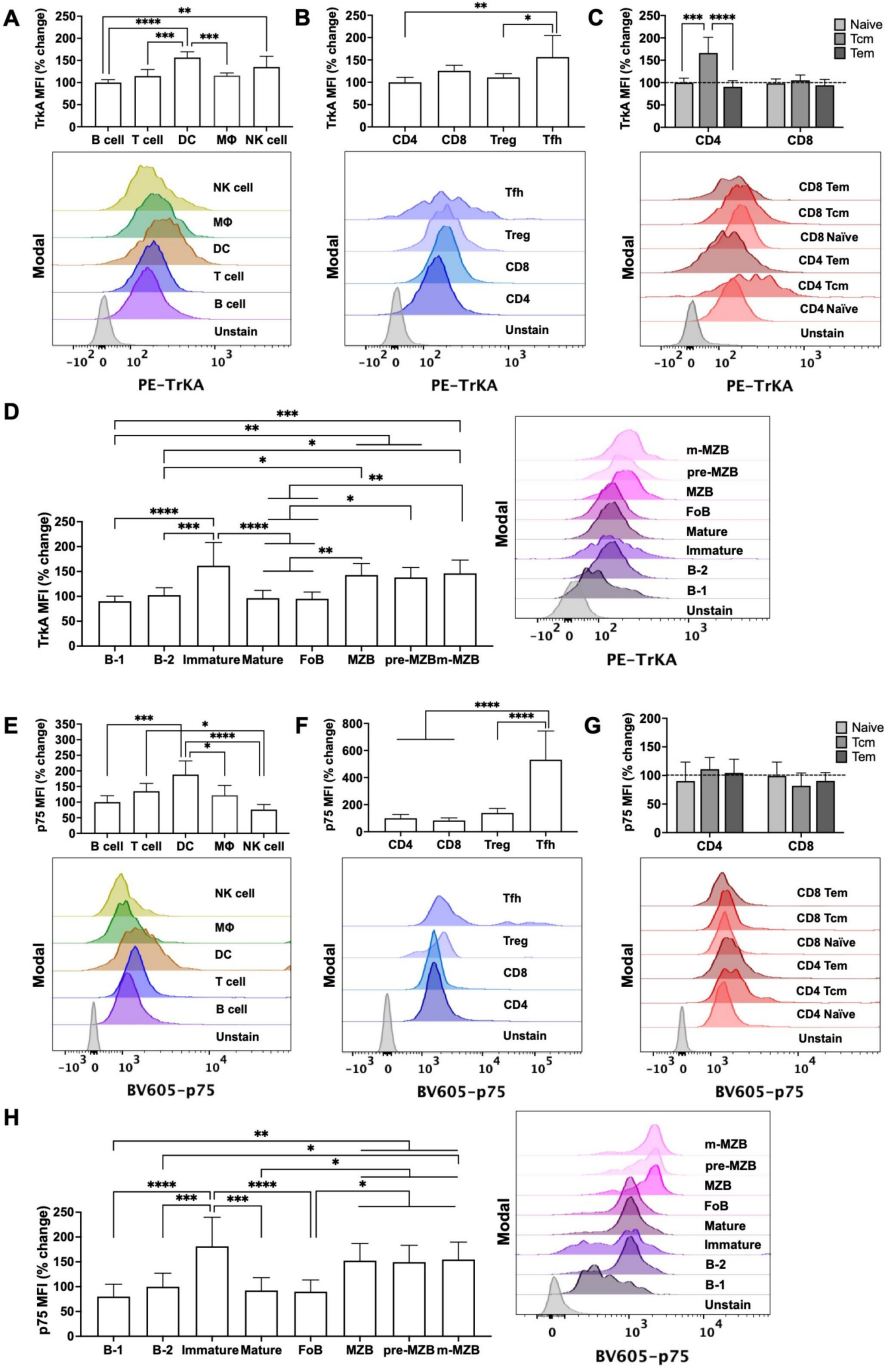
Expression of NGF receptors (TrkA and p75) on immune cells. Bar plots present comparative changes in mean fluorescence intensity (MFI) of TrkA and p75 among all the examined immune cell types. Differential expressions of TrkA and p75 among immune cells was calculated as percent change compared to MFI of TrkA and p75 in B cells **(A, E)**, compared to MFI of TrkA and p75 in CD4^+^ T cells **(B, F)**. Percent change in TrkA MFI **(C)** and p75 MFI **(G)** among naive and memory T cell (Tcm and Tem) subsets of CD4 and CD8 lineage was calculated by comparing with TrkA and p75 MFI in total CD4 and CD8 T cell population (presented as dotted line). Percent change in TrkA and p75 MFI among B cell subsets was calculated by comparing with MFI of TrkA and p75 in B-2 subset **(D, H)**. Representative half-offset histograms present qualitative expression of TrkA and p75 in stained cells against the unstained group **(A–H)**. Data are represented as mean ± S.D. and the statistical comparison across different cell types/subtypes (n=5–8 mice) were analysed by one-way ANOVA with Tukey’s correction for multiple comparisons; *p < 0.05, **p < 0.01, ***p < 0.001, **** p < 0.0001. DC, Dendritic cell; NK cell, Natural killer cell; Mϕ, macrophage; T_reg_ cell, regulatory T cell; T_fh_ cell, follicular T helper cell; T_cm_ cell, central memory T cell; T_em_ cell, effector memory T cell; FoB cell, follicular B cell; MZB cell, marginal zone B cell; pre-MZB cell, precursor marginal zone B cell; and m-MZB cell, mature marginal zone B cell.

Differential expressions of NGF receptors TrkA and p75 were observed among immune cells from innate and adaptive immunity in unchallenged mouse splenocytes. Dendritic cell (DC) expressed significantly higher TrkA levels than B cells, T cells, or macrophages. T cells and B cells expressed similar level of TrkA while the expression in natural killer (NK) cells was comparable to DC (**Figure 1A**). Similarly, the p75 receptor expression was also higher in DC among all the immune cells while NK cells had lower cellular content of p75 among all the analyzed cell types (**Figure 1E**). Among T cell subsets, TrkA and p75 level were higher in follicular T helper (T_fh_) cells than in the other subsets of T cells (**Figures 1B, F**). Among naive and memory T cells, CD4^+^ central memory T (T_cm_) cells expressed significantly more TrkA than CD4^+^ naive and CD4^+^ effector memory T (T_em_) cells while CD8^+^ T_cm_ cell expressed comparable TrkA level as in CD8^+^ naive and CD8^+^ T_em_ cells, respectively. No significant differences in p75 levels among naive, T_cm_ and T_em_ cell from the CD4 and CD8 lineage were observed (**Figures 1C, G**). Differential expressions of NGF receptors were also evident in subsets of B cell (**Figures 1D, H**), where immature B cells were found to show higher NGF receptors expression than mature B cells. Analyses of subtypes of mature B cells i.e. the follicular B (FoB) cells and the marginal zone B (MZB) cells, showed that MZB cells exhibited higher NGF receptors expression than FoB cells. On the other hand, both the subsets of MZB cells (pre-MZB and m-MZB) showed similar level of NGF receptors expression. The gating strategy utilized to identify the wide range of immune cells across the adaptive and innate immunity landscape is shown in **Supplementary Figure 1**, and the percent distribution of all the examined immune cells in the mouse spleen is shown in **Supplementary Figure 2**.

### 3.2 Transcriptomic landscape of neurotrophin-associated molecules in immune cells

**Figure 2:**
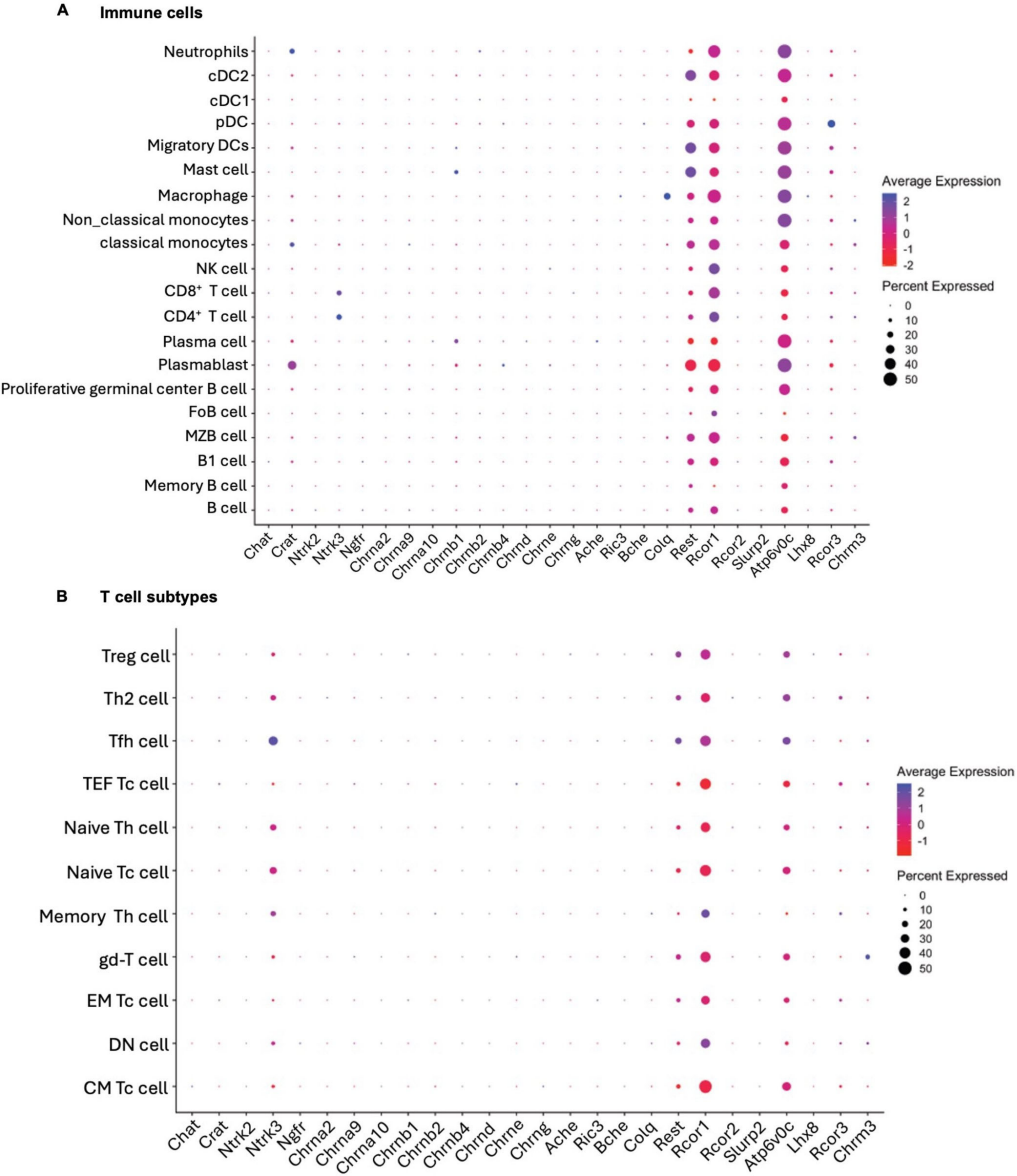
Comprehensive scRNA-seq data for cholinergic and neurotrophin-associated molecules expression in mouse splenic immune cells. Dot plots present expression levels of several cholinergic and NGF-related markers across all the clusters identified as different immune cells **(A)** and sub-clusters from CD3^+^ T cells identified as subtypes of T cells **(B)** collected from spleen of mice (n=2 mice). Average expression indicates the average fold expression levels of the indicated marker in all the cell types. Percent expressed, indicates the percentage of cells in each subcluster where the gene marker is expressed. cDC1, classical type 1 dendritic cell; cDC2, classical type 2 dendritic cell; pDC, Plasmacytoid dendritic cell; NK cell, Natural killer cell; FoB cell, Follicular B cell; MZB cell, Marginal-zone B cel; CM T_c_ cell, central memory cytotoxic T cell; DN T cell, Double negative T cell; EM T_c_ cell, Effector memory cytotoxic T cell; gd-T cell, gamma delta T cell; Memory T_h_ cell, Memory T helper cell; Naive T_c_ cell, Naive cytotoxic T cell; Naive T_h_ cell, Naive T helper cell; TEF T_c_ cell, Terminal effector memory cytotoxic T cell; T_fh_ cell, follicular T helper cell; T_h_2 cell, T helper 2 cell; T_reg_ cell, regulatory T cell.

We next asked whether unchallenged immune cells express components associated with cholinergic as well as NGF signaling, since NGF is known to induce ACh production and associated signaling pathways (19). In-depth analysis of all the identified splenic immune cells and T cell subsets by scRNA-seq showed heterogeneity in the expression profile of several genes associated with neurotrophin signaling and cholinergic signaling (**Figures 2A, B**). We did not find gene expression of *NTRK1* (TrkA) while the expression of *NGFR* (p75) is minimal among immune cells from spleen, which has been crosschecked with other gene expression platforms including cellxgene and PanglaoDB (25, 26). Components of the cholinergic signaling analyzed include ACh synthesizing enzymes- *CHAT* and *CRAT* (carnitine acetyltransferase), subtypes of ACh receptors (nicotinic: nAChR’s and muscarinic: mAChR’s), nAChR ligand- *SLURP 2* (secreted mammalian Ly-6/uPAR-related protein-2), chaperone of AChR- *RIC3*, mediatophore involved in ACh release- *ATP6V0C*, regulator of ChAT expression- *REST* (RE1-silencing transcription factor), corepressors of REST- *RCOR 1, RCOR 2* and *RCOR 3*, ACh degrading enzymes- *ACHE* and *BCHE*, and mediators of NGF signaling include neurotrophin receptors (*NTRK2, NTRK3, NGFR*), and *LHX8* (LIM homeobox 8).

Heterogenous expressions of *RCOR1* and *REST* were found among all the examined immune cell types listed in **Figure 2** except cDC1 (classical dendritic cells 1) and memory B cells for both the genes and FoB cells for *REST* gene. Distinguished expression of *RCOR3* was observed only in pDC (plasmacytoid dendritic cell) and *CRAT* gene expressed in plasmablasts, classical monocytes and neutrophils. mRNA expression of neurotrophin receptor *NTRK3* (TrkC) observed only in CD4^+^ and CD8^+^ T cells with prominent expression recorded in T_fh_ cells among all the subsets of T cells. Some observable expressions of *CHRNB1* mRNA found in minor percentage of mast cells and plasma cells. Differential expressions of *ATP6V0C* were also evident among all the immune cell types except FoB cells, a subtype of B cells and memory T_h_ cells.

The annotation markers for the identification of main clusters as well as the subclusters of T cell are presented as heatmap in **Supplementary Figure 3**.

### 3.3 Immune cell activation can modulate NGF associated markers

**Figure 3:**
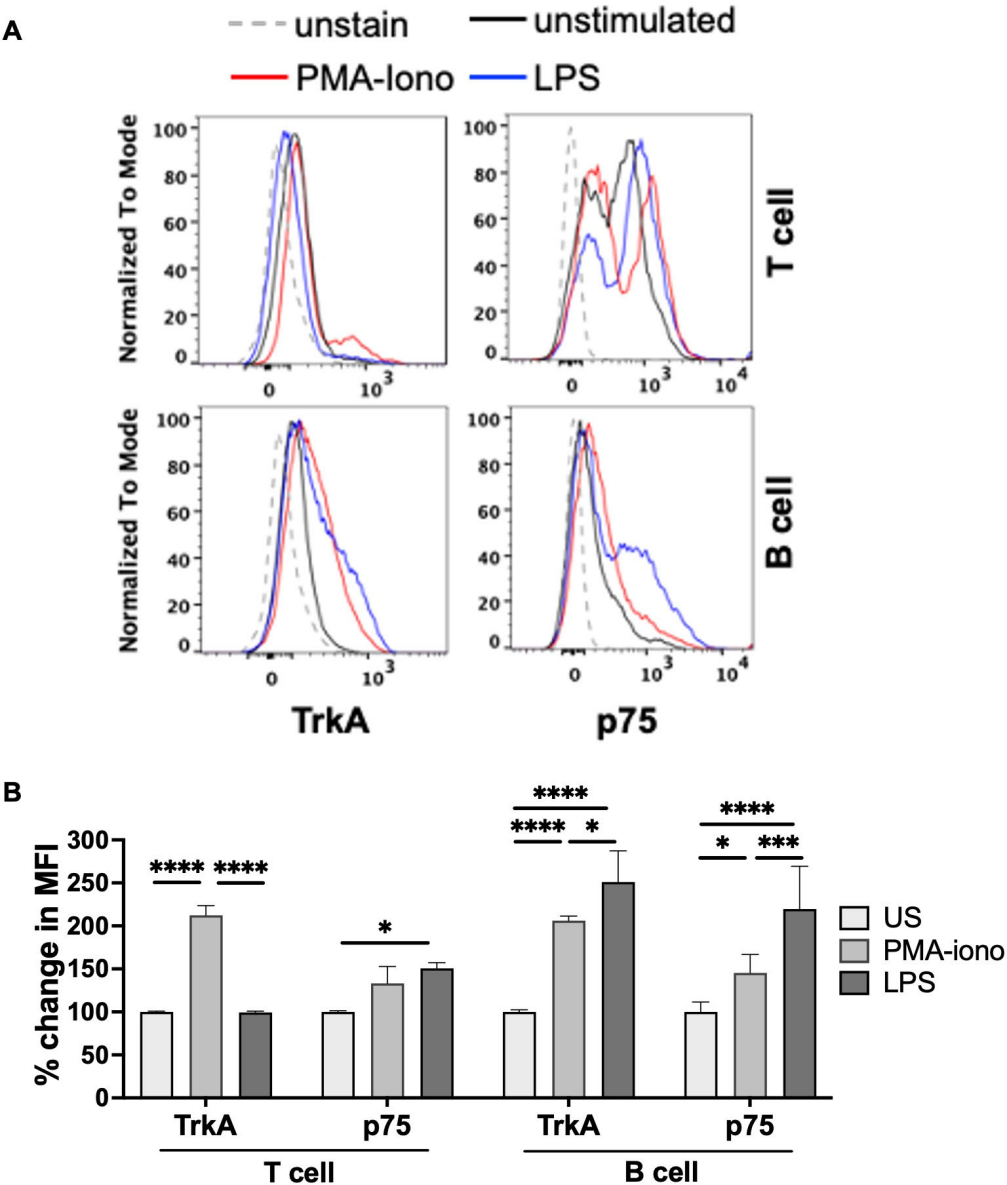
Changes in NGF receptors expression upon splenocyte activation: Spleen dissected from wild type C57BL/6J mice were processed to isolate splenocytes and plated as 1 million cells per well in the 96-well plate. After 48hr stimulation either with PMA and iono (PMA:100 ng/ml; iono: 2 μg/ml) or LPS (1 μg/ml), splenocytes were examined for TrkA and p75 by flow cytometry. **(A)** Histograms present expression of TrkA and p75 in unstimulated (black line), PMA-iono treated (red line) or LPS treated (blue line) T (CD3^+^ gated) and B (B220^+^ gated) cells. Unstained controls were displayed as grey dotted line. **(B)** Bar plot presents percent change in mean fluorescence intensity (MFI) of TrkA and p75 in stimulated groups (PMA-iono or LPS) compared to respective unstimulated (US) group in T (CD3^+^ gated) and B (B220^+^ gated) cells. Data are represented as mean ± S.D. and the statistical comparison across different groups was analysed by two-way ANOVA with Tukey’s multiple comparisons test (three independent experiments; n=3 mice); *p < 0.05, ***p < 0.001, ****p < 0.0001.

To further understand their biological significance, the effect of activation of splenic immune cells by Phorbol myristate acetate (PMA) and ionomycin (iono) or LPS on the expression of NGF receptors in lymphocytes was investigated (**Figures 3A, B**). PMA-iono significantly increased TrkA levels in both T and B cells, with significant p75 increase observed only in the B cells compared to unstimulated T and B cells. On the other hand, LPS mediated activation of splenocytes lead to significantly elevated p75 levels in T and B cells with significant TrkA induction observed in B cells alone when compared to respective unstimulated T and B cells (**Figure 3B**).

### 3.4 NGF treatment reduced TNF-α cytokine secretion from splenic T-and B cells

**Figure 4:**
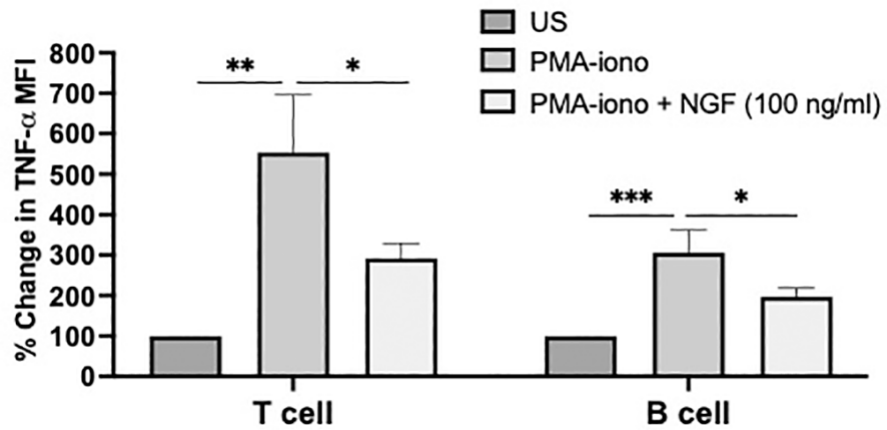
Effect of NGF treatment on cytokine production in splenocytes. Spleens dissected from wild type C57BL/6J mice were processed to isolate splenocytes and plated in 96 well plate with 1 million cells per well. Splenocytes were treated with either PMA-iono (PMA:100 ng/ml; iono: 2 μg/ml) or PMA-iono supplemented with 100 ng/ml mature-NGF for 48hr and examined for TNF-α cytokine expression in CD3^+^ T cells and B220^+^ B cells by flow cytometry. The bar diagram presents percent change in mean fluorescence intensity (MFI) of TNF-α in stimulated groups (PMA-iono or PMA-Iono + NGF) compared to respective unstimulated (US) group in T (CD3^+^) and B (B220^+^) cells. Data are represented as mean ± S.D. and statistical comparison across different groups (performed as three independent experiments, n=3 mice) were analysed by one-way ANOVA with Tukey’s correction for multiple comparisons; *p < 0.05, **p < 0.01, ***p < 0.001.

To further understand the role of NGF-induced signalling in immune cells function, activated splenic immune cells were exogenously supplemented with mature NGF and the secretion of inflammatory cytokine TNF-α was examined in T and B cells by flow cytometry. We found that PMA-iono mediated stimulation increased 5.54-fold TNF-α production in T cells and 3.06-fold in B cells compared to the unstimulated T and B cells, respectively (**Figure 4**). However, treatment with NGF significantly reduced PMA-iono induced TNF-α production to 2.92-fold in T cells and 1.97-fold in B cells.

### 3.5 Hampered expression of NGF-receptors in a mouse model of AD with progressive Aβ pathology

**Figure 5:**
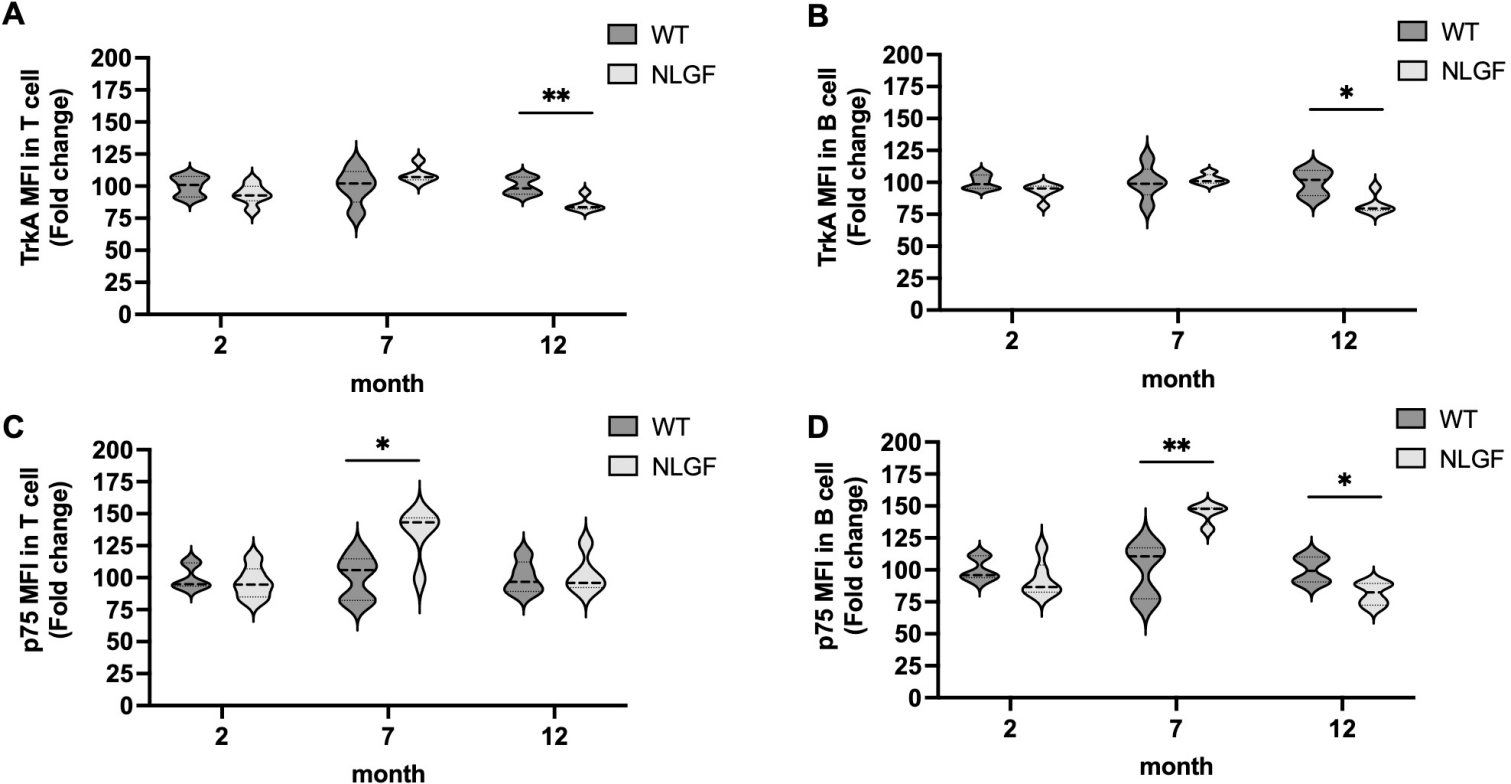
Changes in NGF receptors (TrkA and p75) expression in mouse splenic T and B cells in AD continnum. Splenocytes collected from the spleen dissected from wild type (WT) and AD mouse model (NLGF) were examined at 2, 7 and 12-months of age. Violin plots present fold change in mean fluorescence intensity (MFI) of TrkA in CD3 gated T cells **(A)** and B220 gated B cells **(B)** as well as p75 in CD3 gated T cells **(C)** and B220 gated B cells **(D)** of NLGF mice compared to WT mice (n = 4–6 mice per genotype). Data are represented as mean ± S.D. and the statistical comparison between WT and NLGF group was analyzed by unpaired *t-*test; *p < 0.05, **p < 0.01.

We utilized *App^NL-G-F^* knock-in mouse model (NLGF) which develops Aβ plaques in the brain from the age of 2 months onwards, with cognitive deficits from 6 months onwards (24). This is a model of typical Aβ pathology, neuro-inflammation, and memory impairment in an age-dependent manner. Changes in NGF receptors (TrkA and p75) expression in splenic CD3^+^ T and B220^+^ B cells from wild-type (WT) and NLGF mice at 2, 7, and 12 months of age were examined (**Figure 5**). The level of p75 was significantly increased in T and B cells from NLGF mice at 7-month age with no significant alterations in TrkA compared to levels in age matched WT mice. In contrast, significant reduction in TrkA level in T and B cells in 12-month-old NLGF mice compared to WT mice were found. In addition, a significant decrease in p75 levels was observed in B cells in 12-month-old NLGF mice. Similarly, among innate immune cells from NLGF mice, elevated level of p75 was also evident in DC, macrophage and NK cells at 7-month of age with significant decrease in p75 in DC and TrkA in macrophage at 12-month age compared to age-matched WT mice (**Supplementary Figure 4**).

## 4 Discussion

This study reports the existence of NGF-receptors (TrkA and p75) on various immune cells from innate and adaptive immunity. We also report that immune cell activation can modulate the expression of NGF receptors. Our analysis reveals that NGF treatment reduced inflammatory cytokine production from immune cells, indicating the crucial role of NGF mediated signaling on immune cells. We report alterations in NGF-receptors expression in immune cells in an age-dependent manner in an AD mouse model, providing plausible explanation for increased chronic inflammation in the AD condition.

NGF is the founding member of the neurotrophin family, and its neurotrophic effect is well-studied in the case of neurons (19). Under normal conditions, it is released extracellularly as proNGF (precursor) which is metabolized to mNGF (mature) which interacts primarily with TrkA to induce cell survival pathways. However, when NGF metabolism is compromised, proNGF may preferably bind to p75 to induce cellular dysfunction and death in case of neurons (27). Although NGF-TrkA mediated biological effects were discreetly reported in few immune cell types (17, 28), a comprehensive examination of the presence of NGF-associated components in the immune cells are for the first time provided in this study. Our study provides evidence that each major type of immune cells including DC, macrophage, NK cells, as well as various subtypes of lymphocytes (B and T cells) possesses both the NGF-receptors - TrkA as well as p75, which thus classifies all these immune cells as responsive to both the forms of NGF (proNGF and mNGF) (**Figure 1**).

Comparative analysis among all the examined immune cells revealed that DC expresses higher levels of TrkA and p75 than other immune cells including macrophage, NK cells, and lymphocytes (**Figure 1**). Higher expression of NGF receptors in DC is a subject of interest because DC serve as a bridge between innate and adaptive immune system (29). Among memory and naive CD4^+^ T cells, CD4^+^ T_cm_ cells expressed more TrkA than the naive counterpart indicating towards possible involvement of TrkA receptor in modulating immune response during antigen challenge. Higher TrkA expression on memory T cells may enhance their ability to respond to NGF, which may support prolonged survival, activation, and migration of memory T cells, which needs further research. We also gained first-hand evidence that immature B cells express more NGF receptors than mature B cells, emphasizing towards the role of these receptors in B cell maturation and differentiation (30). Similarly, MZB cells exhibited higher NGF receptors expression than FoB cells indicating towards differential requirements of NGF by them.

We also performed comprehensive mapping of gene expression for cholinergic and NGF-associated components among all types and subsets of immune cells (**Figure 2**). We observed heterogeneity in the expression of ChAT regulator *REST and RCOR1* as well as the receptor *NTRK3* (TrkC) mRNA among all types and sub-types of immune cells. We also report on the existence of mediatophore *ATP6V0C* in the immune cells and their subtypes, wherein *ATP6V0C* is reported to be involved in the secretion of ACh from the cells (31). Expression of *ATP6V0C* and its role in the release of ACh in two different human T cell lines was previously reported (32). The observed heterogeneity in the expression of these genes among immune cells and their subtypes reflects that all these immune cells may exhibit differential response upon receiving comparable cholinergic input. Our scRNA-seq data confirms previous findings that the mouse immune cells do not express ChAT mRNA under resting condition (33). Interestingly, we have observed a discrepancy between gene and protein expression for TrkA (*NTRK1*) and p75 (*NGFR*). While the protein levels were detectable with flow cytometry, the gene expression data was often below the threshold for detection, likely due to a combination of low turnover of the NGF receptors (and thus, low gene expression), and the relative stochastic detection of mRNA transcripts by scRNA-seq. However, our observations are supported by studies confirming the protein expression of NGF receptor p75 on DC, mast cells and B cells as well as TrkA receptor expression in macrophages, mast cells, and lymphocytes (16, 17, 34, 35). Interestingly, previous studies utilizing a reporter mouse (ChAT^BAC^-eGFP transgenic mice) expressing GFP under the control of the ChAT promoter (ChAT-eGFP) (36), have found ChAT protein expression in T-cells (4.4% of CD4^+^CD8^-^) (37), and B-cells (11% follicular and 18% of marginal zone) (7), however we have not found any gene expression for ChAT in our dataset, and neither in the publicly available datasets (25, 26).

Our study provides the first evidence that the expression of NGF receptors is dynamically controlled during activation of T and B cells by PMA-iono or LPS, where the stimulants involve different routes of signalling (**Figure 3**). Differential effects on the induction of TrkA and p75 in T and B cells were observed by PMA-iono and LPS. One possible explanation is that activation by PMA-iono is cell type-independent, while LPS interacts with the TLR4 receptor (which is more highly expressed in B cells than in T cells) and initiates cell type specific signaling (38, 39). However, this response could also be cell type and time dependent variables, which invites further research. Overall, enhanced TrkA expression in T and B cells suggested towards the functional significance of NGF-TrkA signaling in both the cell types during/after activation or possibly for inflammation resolution. The later possibility would be interesting since mature NGF could induce an anti-inflammatory effect on T and B cells (**Figure 4**), possibly indicating towards a feedback mechanism which may control eventual resolution phase post activation. These speculations need further research.

The dynamics of NGF metabolism and the concomitant expression of NGF-receptor subtypes (TrkA or p75) may dictate the overall effect on cells. The altered TrkA/p75 ratio and hampered NGF metabolism have been suggested to lead to degeneration of cholinergic neurons in AD (19). AD has been previously reported to represent chronic inflammation and NGF dysmetabolism in the brain and peripheral system (21, 22). However, whether the NGF receptors expression are affected in immune cells in case of AD was not known. Our study provides first evidence on the loss of NGF-receptors expression in T and B cells in an AD mouse model of Aβ pathology (**Figure 5**), indicating towards altered NGF-immune signaling in AD. Surprisingly, the expression of p75 in T and B cells increased in 7-month-old mice while the expression of TrkA was found to be unchanged in 7-month-old mice and was found reduced in 12-month-old mice. Although increased p75 expression in 7-month-old mice coincide with the initiation of altered cognition in this mice model (24), any correlation between p75 expression and cognition needs further exploration. Irrespective of the alterations in p75 and TrkA expressions at different time points, the TrkA/p75 ratio in T and B cells remained altered starting from 7-months and onwards (**Supplementary Figure S5**). This may provide a plausible link between immune cells-induced chronic inflammation and hampered NGF metabolism in AD, which have been individually reported in AD patients (21, 22). Our study indicates that the source of chronic inflammation in AD may at least in part be due to loss of NGF-mediated anti-inflammatory effects in immune cells. Although reduced NGF-TrkA signaling has been reported to induce loss of cholinergic phenotype in neurons previously (19, 40), the biological impact of reduced TrkA expression on immune cells (as observed in the AD mice model: **Figure 5**) or reduced availability of mNGF in AD patient plasma (22), needs to be ascertained in future studies. Indication can be found from a different condition where immune cells from patients with juvenile idiopathic arthritis displayed reduced TrkA expression compared to healthy controls suggesting that defective NGF receptor expression may facilitate inflammatory mechanisms (14). Likewise, low plasma level of NGF in HIV-1 infected patients were associated with death of memory B cells thus suggesting the possible contribution of NGF towards B cell survival (41). We report NGF-receptor expressions in all subtypes of B cells (**Figure 1**).

NGF receptor mediated signaling has been directly involved in modulating function in certain immune cells. p75 receptor can modulate the ability of DC to stimulate T cells along with DC-mediated allergic asthma in mice model (28, 42). This may explain how NGF can regulate the immune response by modifying the immune cell’s function. We also show that NGF-receptors are biologically active and regulate inflammatory cytokines production ex-vivo (**Figure 4**) signifying their biological relevance in T and B cell function. Although future studies are needed to understand the physiological role of NGF receptors on each of these immune cell subtypes, in various aspects including cell development, differentiation and function. We provide evidence that NGF-mediated stimulation can certainly counteract inflammatory reactions from T and B cells, supported by previous study on human monocytes (14). Our findings highlight the need to reconsider the present view that anti-inflammatory effects on immune cells are primarily driven by cholinergic receptors (43). Instead, we propose that under physiological conditions NGF-mediated pathways maintain anti-inflammatory effects in immune cells due to the widespread presence of NGF receptors on immune cells and its ligand NGF in the tissues and blood/plasma (44). These interactions provide a direct peripheral neuro-immune regulatory network, beyond or in addition to the cholinergic control of immune function since ACh is susceptible for quick clearance by cholinesterase’s (45, 46). Furthermore, NGF-mediated stimulation of immune cells may induce ACh production (a feature of cholinergic neurons) representing a feed-forward loop, as shown in the graphical abstract. Interestingly, few immune cells were previously reported to produce NGF (47), which further indicates the close association of NGF-cholinergic-immune crosstalk, and lends explanation how immune cells themselves play an active role in inflammation resolution (48).

Although NGF and the cholinergic pathways are closely linked, their interaction in the context of immune cells are not yet fully understood. Significant amount of literature exists about the presence of cholinergic receptors (muscarinic – mAChR’s; and nicotinic – nAChR’s) on few immune cell types under *in-vivo* or ex-vivo conditions along with ACh content (reviewed by Fujii et al.) (49). Various other groups have shown biological significance of lymphocyte-derived ACh on various aspects like immune function (7), neuroimmune communication via the vagus nerve dependent pathways in T-cells (37) or vagus nerve independent pathways in B-cells (7), clearance of infectious pathogens and blood vessel modulation (9). Recent reports further show that certain parts of the CAIP are dispensable and not dependent on lymphocyte relay altogether (50), which may need further attention to refine our understanding of the role of central pathways in regulating peripheral inflammation. It should also be noted that the exact role of cholinergic function on all immune cell sub-types is far from being understood.

Our study has important implications on various aspects of immunobiology and inflammation in physiological and pathological contexts. This study opens the possibility to pharmacologically target TrkA mediated signaling in immune cells to modulate inflammation, and perhaps others like metabolism, antibody production, immune cell survival and differentiation, etc. Interestingly, it was recently shown that NGF stimulation may modulate certain aspects of cholesterol metabolism (51, 52) which can possibly reprogram the metabolic pathways within certain cells. Now that we know the presence of TrkA on many other immune cell types and subtypes, targeting immune metabolism could be a crucial aspect in anti-inflammatory strategies as previously reviewed (53). Furthermore, recent studies have shown that resolution of inflammation is not the end of immunological activity but perhaps shape future immune physiology of tissues (5), wherein the role of NGF and cholinergic pathways is important and needs to be investigated in depth, which is intriguing due to enhanced expression of NGF-receptors on antigen challenged cells (**Figure 1** and **Figure 3**).

### 4.1 Strengths and limitations of the study

We have used two mouse models (wildtype and AD) to validate our results, showing protein expression of NGF receptors in various types and subtypes of immune cells. This animal data may or may not show differential results from their human counterparts, making studies on human tissues the next step. To try to mimic the human situation in AD as much as possible, we chose a humanized AD mouse model which develops Aβ pathology along with inflammation and cognitive impairment. However, it does not reflect tau and neurodegeneration, which are additional features of AD, thus not including the full AD phenotype in the model. We chose this model since the pathology is purely driven by amyloid, which is now actively targeted for disease-modifying therapies. From a technical perspective, we have used antibodies to detect proteins however they cannot differentiate between splice variants or other types of variants arising from posttranslational modifications or signaling pathways (e.g., phosphorylation). Use of scRNA-seq has its own limitation of detecting transcripts which are expressed above a cutoff level and express a high number of reads; thus, it does not detect gene products which are below cutoff levels or low in number of reads, which is further complicated by the differential regulation at transcriptional and translation levels. The strength of using unchallenged animal models is to show the basal level of gene expression on various cells; however, it may not depict the presence of gene expression which maybe present on activated immune cells.

## Data Availability

The data presented in the study is deposited at DORIS - Swedish National Data Service. The link is provided below: https://doi.org/10.48723/d5mw-0122.
