# Supplementary material for: Nerve growth factor responsive elements modulate immune cell inflammation and are dysregulated in an Alzheimer’s disease mouse model
Source: Front Immunol. 2026 Apr 23;17:1722477. doi: 10.3389/fimmu.2026.1722477 (PMC13149181; doi:10.3389/fimmu.2026.1722477)
Supplement: Supplementary file 1 [file DataSheet1.pdf]

Nerve growth factor responsive elements modulate immune cell inflammation and are dysregulated in an Alzheimer's disease mouse model

Ruchi Gera[1*], Ph.D., Giuseppe Mocci[2], Ph.D., Simone Tambaro[3], Ph.D., Michael Vanlandewijck[2,4], Ph.D., Per Nilsson[3], Ph.D., Maria Eriksdotter[1,5†], MD, Ph.D., Sumonto Mitra[1†], Ph.D.

Affiliations:

[1] Department of Neurobiology, Care Sciences and Society, Division of Clinical Geriatrics, Center for Alzheimer Research, Karolinska Institutet, 141 52 Huddinge, Sweden.

[2] Department of Medicine, Huddinge, Karolinska Institutet, 141 52 Huddinge, Sweden

[3] Department of Neurobiology, Care Sciences and Society, Division of Neurogeriatrics, Center for Alzheimer Research, Karolinska Institutet, 171 64 Stockholm, Sweden.

[4] Department of Immunology, Genetics and Pathology, Uppsala University, 751 85 Uppsala, Sweden

[5] Theme Inflammation and Aging, Karolinska University Hospital, 141 86 Huddinge, Sweden

* To whom correspondence may be addressed: Ruchi Gera, Center for Alzheimer Research, Dept. of Neurobiology, Care Sciences and Society (NVS), Division of Clinical geriatrics, Karolinska Institutet, Blickagången 16, NEO plan 7, 141 83 Huddinge, Sweden, Tel.: +46769704400; email- ruchi.gera@ki.se

[†] Co-last/Equal authorship

Running title: Neurotrophin-cholinergic network in immune cells

SUPPLEMENTARY INFORMATION (SI)

Supplementary Figure 1. Gating strategy utilized to identify immune cells

Supplementary Figure 2. Distribution of immune cells in mice spleen

Supplementary Figure 3. Heatmap of marker genes utilized to annotate cell clusters

Supplementary Figure 4. Changes in NGF receptors expression in mouse splenic innate immune cells

Supplementary Figure 5. Changes in TrkA/p75 ratio in mouse splenic immune cells

Supplementary Table 1. List of fluorochrome conjugated antibodies utilized in flowcytometry.

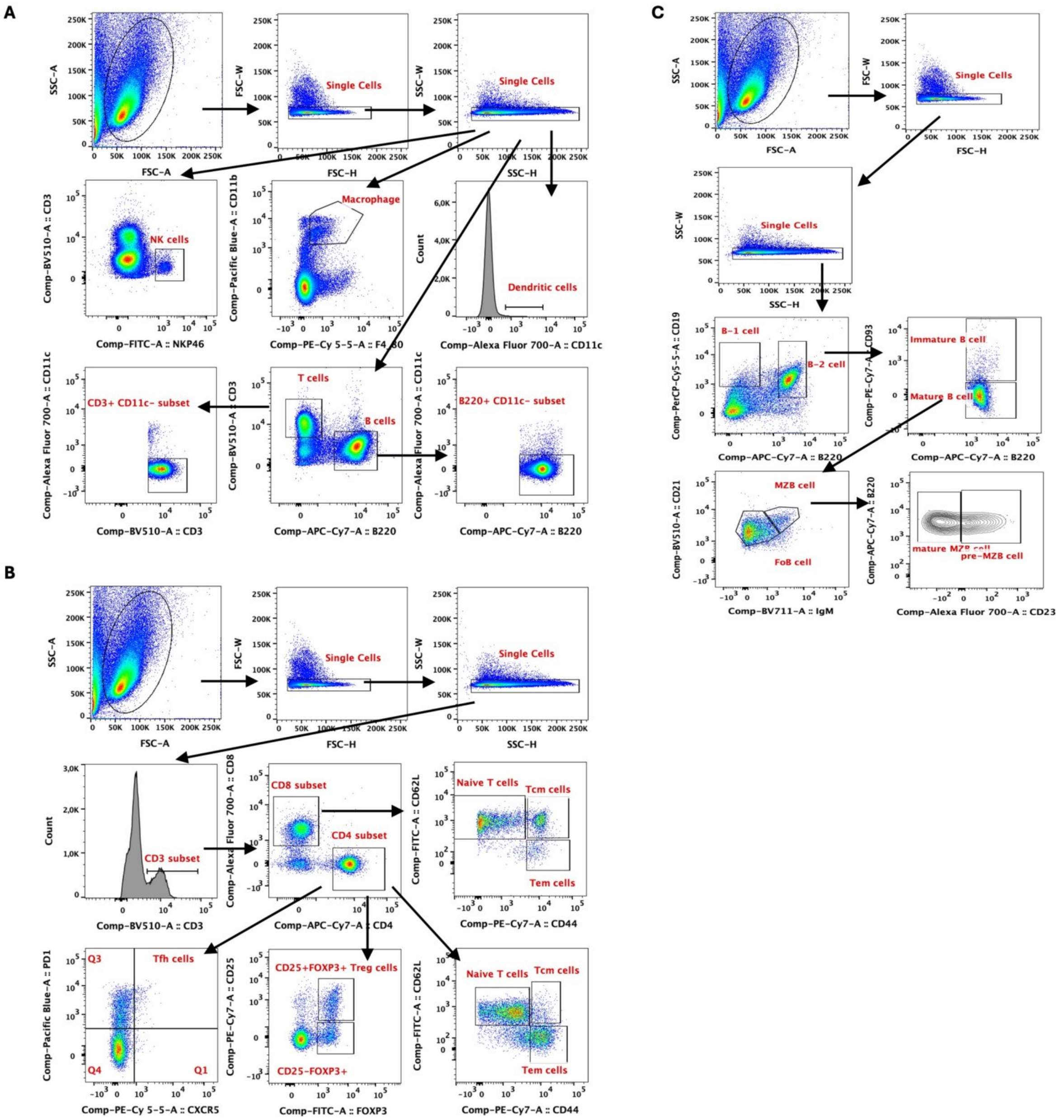

Supplementary Figure 1. Gating strategy utilized to identify immune cells. Stained mouse splenocytes were acquired on LSRII flowcytometer and gated single cells were analyzed to identify splenic immune cell types and sub-types using cell specific determinants. (A) various immune cells were identified as natural killer (NK) cell ($CD3^{-}NKp46^{+}$), Macrophage ($CD11b^{+}$ $F4/80^{+}$), Dendritic cell ($CD11c^{+}$), T cell ($CD3^{+}B220^{-}CD11c^{-}$), and B cell ($B220^{+}CD3^{-}CD11c^{-}$). (B) single cell, size gated splenic cell identified for T cell (CD3+) and T subsets were separated into helper T cell as $CD3^{+}CD4^{+}$ and cytotoxic T cell as $CD3^{+}$ $CD8^{+}$ T cell.

$CD4^+$ T cell subset was further gated for follicular T cell (T_{fh}) cell as $CD3^+CD4^+PD1^+CXCR5^+$ and regulatory T (T_{reg}) cell as $CD3^+CD4^+CD25^+Foxp3^+$. Memory T cell from CD4 and CD8 lineage were classified as T_{em} cell ($CD44^+CD62L^-$), T_{cm} ($CD44^+CD62L^+$) and naive T cell ($CD44^-CD62L^+$). (C) B cell gating includes B-1 and B-2 cell identified as $CD19^+B220^-$ and $CD19^+B220^+$ respectively. B-2 cell were further gated for immature B cell as $CD93^+B220^+$ and mature B cell as $CD93^-B220^+$. Mature B cell was separated into follicular B (FoB) cell as $CD21^{int}$ $IgM^{int/+}$ and marginal zone B (MZB) cell as $CD21^+IgM^+$. Expression of CD23 was further used to separate mature-MZB cell ($CD23^-B220^+$) from precursor-MZB cell ($CD23^+B220^+$). T_{cm} cell: central memory T cell; T_{em} cell: effector memory T cell

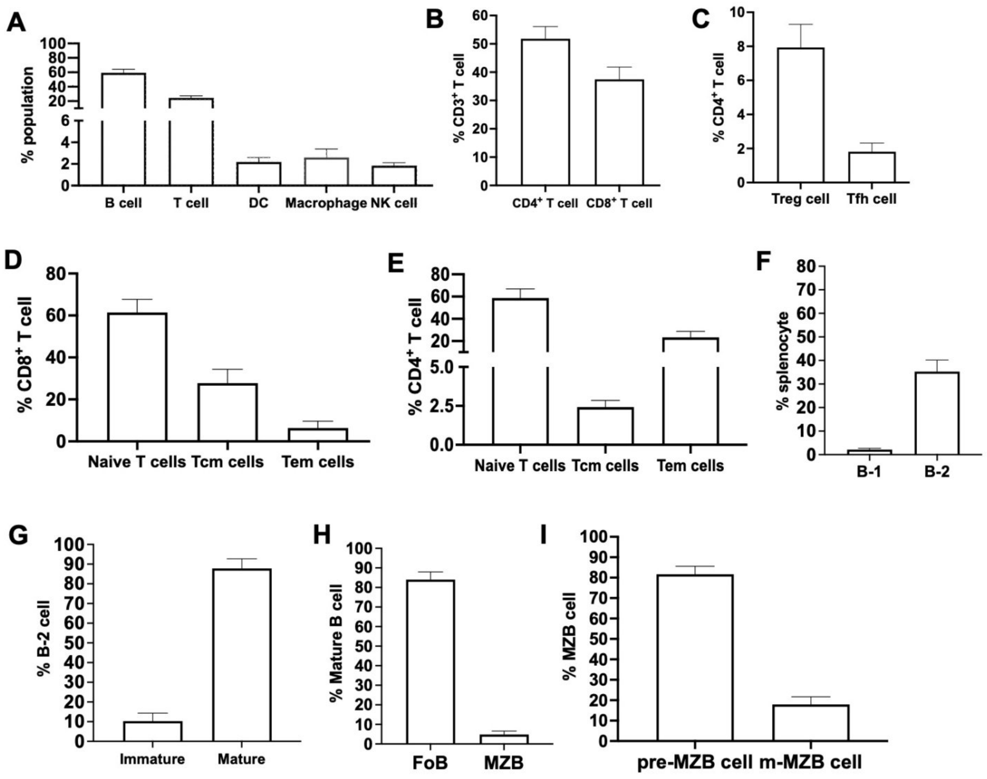

Supplementary Figure 2. Distribution of immune cells in mice spleen. Stained mouse splenocytes were acquired on LSRII flowcytometer and gated cells were analyzed for the composition of immune cells in spleen (n=5-8 mice). Bar plots represent the percentage of B cell, T cell, dendritic cell (DC), macrophage and natural killer (NK) cell in splenocytes (A), percentage of $CD4^+$ and $CD8^+$ T cell in $CD3^+$ gated T cell (B), percentage of regulatory T (T_{reg}) cell and follicular T helper (T_{fh}) cell in CD4 gated T cell (C), percentage of naive T cell, T_{cm} and Tem cell in CD8 gated T cell (D) and CD4 gated T cell (E). Percentage of B-1 and B-2 cell in total splenocyte (F), percentage of immature and mature B cell in B-2 gated cell (G), follicular B (FoB) cell and marginal zone B (MZB) cell in gated mature B cell (H), percentage of precursor marginal zone B (pre-MZB) and mature marginal zone B (m-MZB) cell in gated MZB cell (I). T_{cm} cell: central memory T cell; T_{em} cell: effector memory T cell.

A

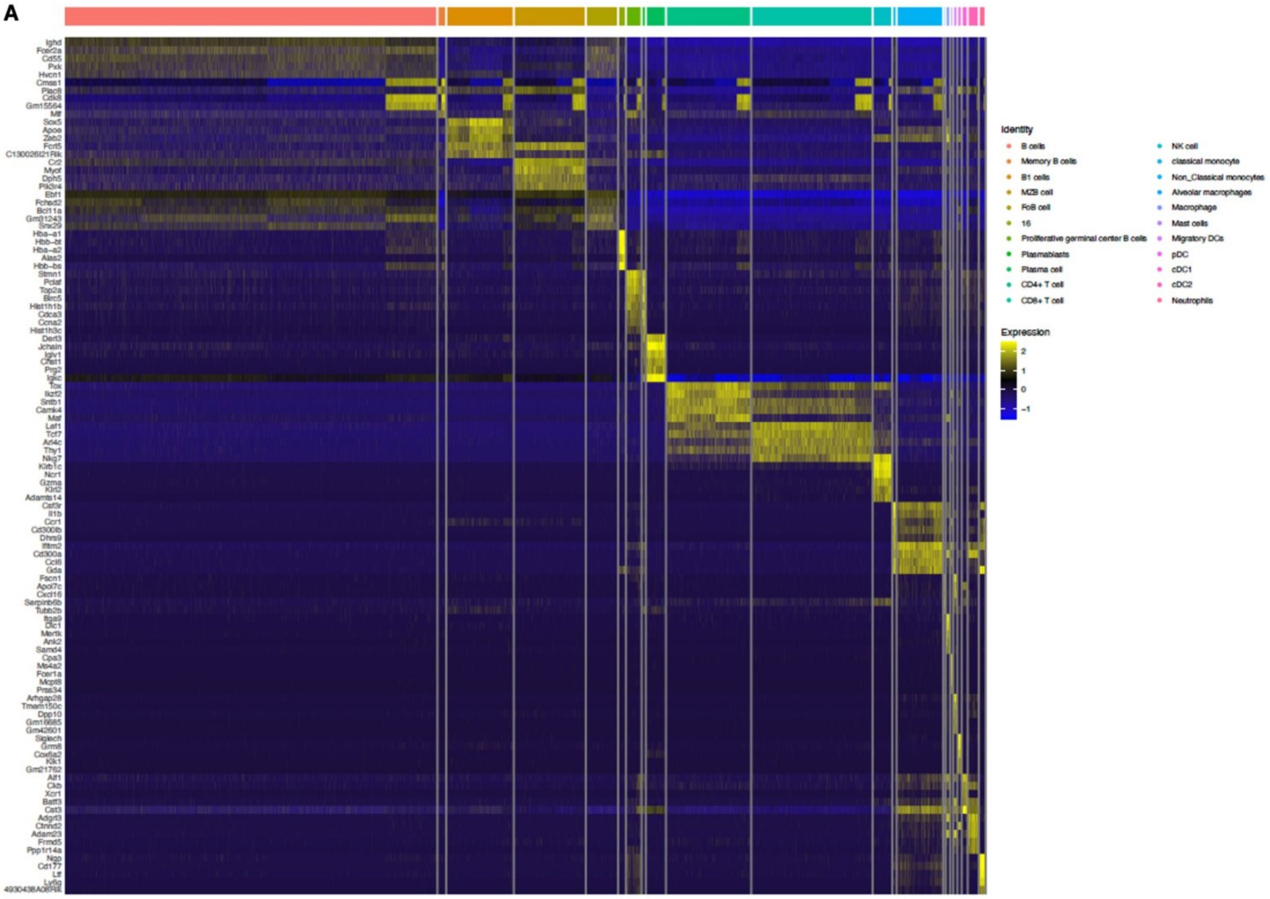
Identity
B cells
Memory B cells
B1 cells
MZB cell
FoB cell
16
Proliferative germinal center B cells
Plasmablasts
Plasma cell
CD4+ T cell
CD8+ T cell
NK cell
classical monocyte
Non_Classical monocytes
Alveolar macrophages
Macrophage
Mast cells
Migratory DCs
pDC
cDC1
cDC2
Neutrophils
Expression
2
1
0
−1

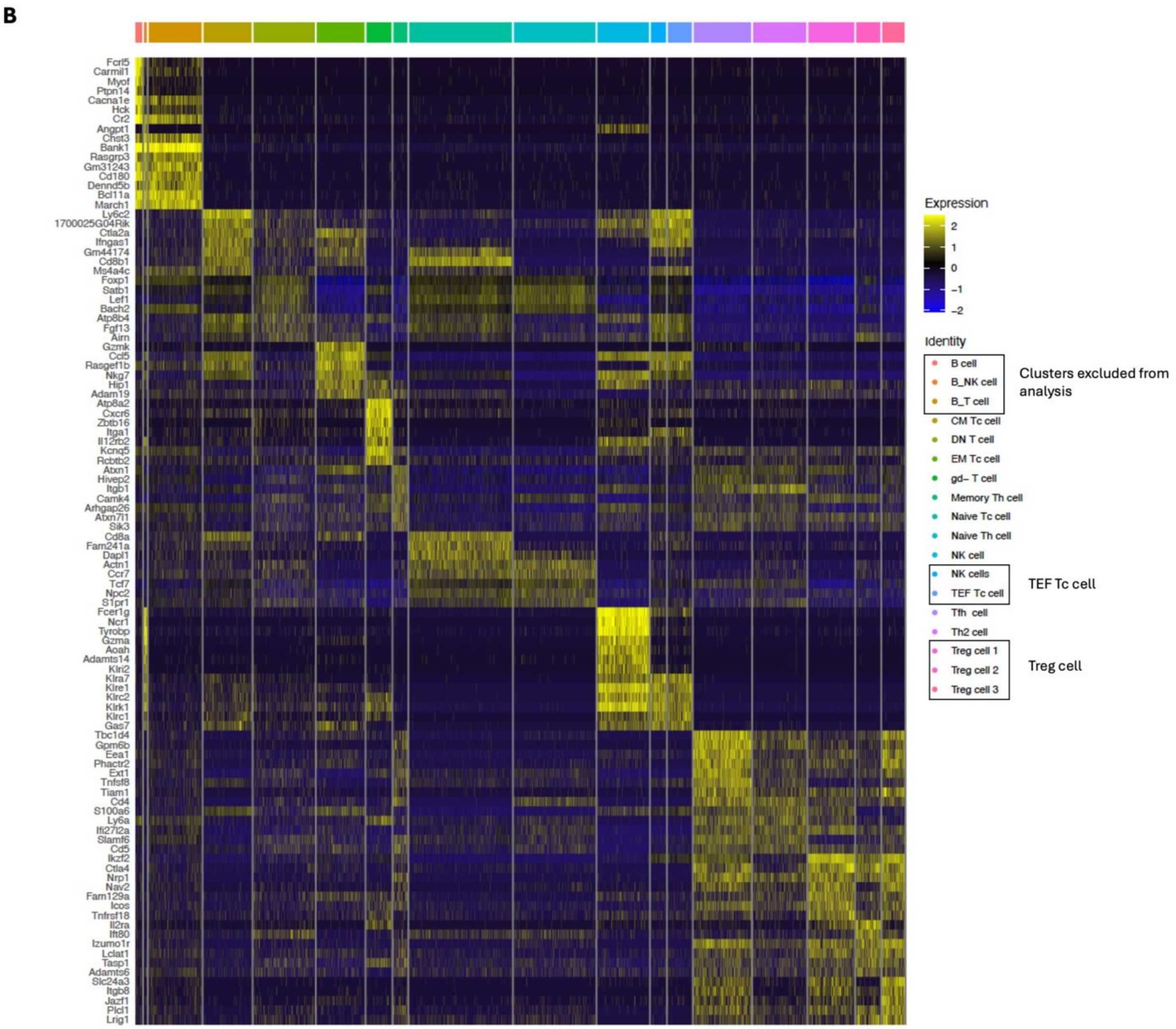

Supplementary Figure 3. Heatmap of marker genes utilized to annotate cell clusters. (A) A heatmap displaying the 5 topmost differentially expressed marker genes used to identify immune cell types. (B) A heatmap from the CD3+ cluster subset from (A), identifying T cell subsets. The top 6 differentially expressed marker genes are displayed. In both graphs, the expression levels are visualized on a log2 scale and differential gene detection was performed using the “vst” method as described in the methods section.

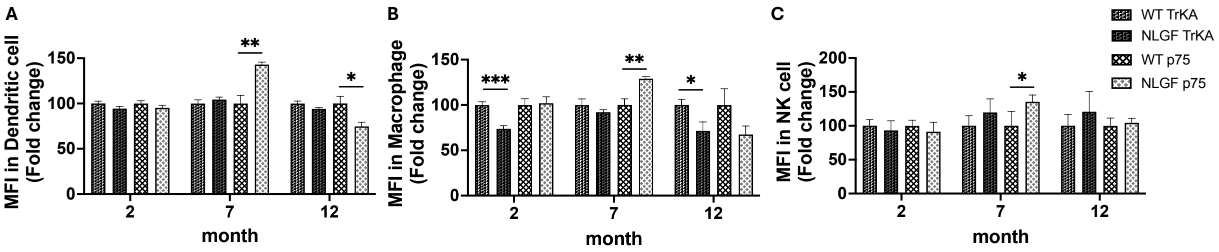

Supplementary Figure 4. Changes in NGF receptors expression in mouse splenic innate immune cells. Splenocytes collected from the spleen dissected from wild type (WT) and AD mouse model (NLGF mice) were examined at 2, 7 and 12 months of age. Bar diagrams present fold change in mean fluorescence intensity (MFI) of TrkA and p75 in $CD11c^{+}$ Dendritic cell (A), $CD11b^{+}F4/80^{+}$ macrophage (B) and $CD3^{-}$ $NKp46^{+}$ NK cell (C) of NLGF mice compared to WT mice (n =4-5 per genotype). Data are represented as mean ± S.D. and the statistical comparison between WT and NLGF group within each time point was analyzed by unpaired t-test; *p < 0,05, **p < 0,01, ***p < 0,001.

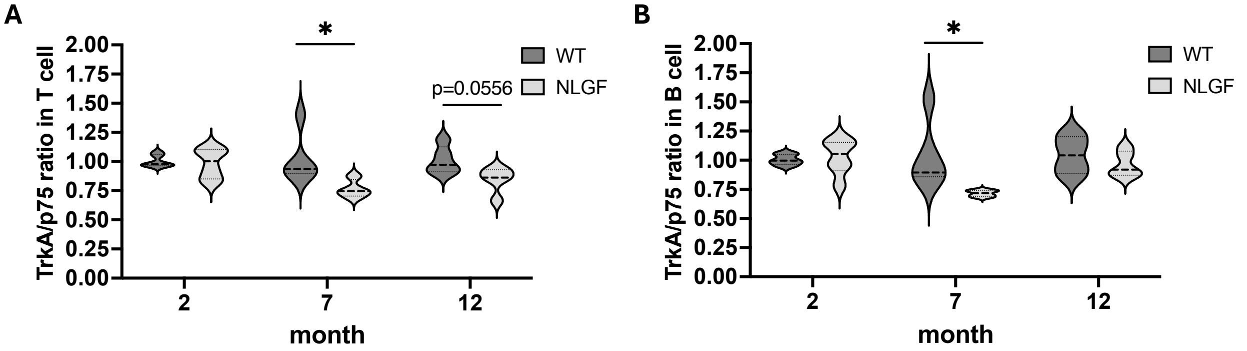

Supplementary Figure 5. Changes in TrkA/p75 ratio in mouse splenic immune cells. Splenocytes collected from the spleen dissected from wild type (WT) and AD mouse model (NLGF) were examined at 2, 7 and 12-months of age. Violin plots present TrkA/p75 ratio in CD3 gated T cells (A) and B220 gated B cells (B) calculated by dividing TrkA MFI (mean fluorescence intensity) fold change by p75 MFI fold change in NLGF mice compared to WT mice (n = 4-6 mice per genotype). Data are represented as mean ± S.D. and the statistical comparison between WT and NLGF group within each time point was analyzed by unpaired t-test using Mann-Whitney nonparametric test ; $^*p < 0.05$

Supplementary Table 1

List of fluorochrome conjugated antibodies utilized in flowcytometry.

Target	Fluorophore	Vendor	Catalogue no.
B220	APC-Cy7	Biolegend	103224
CD11b	Pacific Blue	Biolegend	101223
F4/80	PerCP/Cy5.5	Biolegend	123127
NKp46 (CD335)	FITC	Biolegend	137605
CD11c	AF 700	Biolegend	117319
CD3	BV510	Biolegend	100353
CD4	APC-Cy7	Biolegend	100526
CD8	AF 700	Biolegend	100730
CD25	Pe-Cy7	Biolegend	101916
FOXP3	FITC	Biolegend	320012
PD1 (CD279)	BV421	Biolegend	135221
CXCR5 (CD185)	Percp/Cy5.5	Biolegend	145507
CD62L	FITC	Biolegend	104405
CD44	Pe-Cy7	Biolegend	103029
TNF-a	BV711	Biolegend	506349
TrkA	PE	Abcam	ab209443
CD271 (p75)	Super Bright 600	ThermoFisher	63-9400-42
CD19	PerCP/Cy5.5	Biolegend	115534
CD93	Pe-Cy7	Biolegend	136506
CD21	BV510	Biolegend	123437
IgM	BV711	Biolegend	406539
CD23	AF 700	Biolegend	101632
